# Galeterone and VNPT55 induce proteasomal degradation of AR/AR-V7, induce significant apoptosis via cytochrome c release and suppress growth of castration resistant prostate cancer xenografts *in vivo*

**DOI:** 10.18632/oncotarget.4578

**Published:** 2015-07-14

**Authors:** Andrew K. Kwegyir-Afful, Senthilmurugan Ramalingam, Puranik Purushottamachar, Vidya P. Ramamurthy, Vincent C.O. Njar

**Affiliations:** ^1^ Department of Pharmacology, University of Maryland School of Medicine, Baltimore, MD 21201-1559, USA; ^2^ Center for Biomolecular Therapeutics, University of Maryland School of Medicine, Baltimore, MD 21201-1559, USA; ^3^ Marlene Stewart Greenebaum Cancer Center, University of Maryland School of Medicine, Baltimore, MD 21201-1559, USA

**Keywords:** prostate cancer, androgen receptors (AR/AR-V7), galeterone (gal), gal’s analog VNPT55, mechanisms of AR/AR-V7 degradation

## Abstract

Galeterone (Gal) is a first-in-class multi-target oral small molecule that will soon enter pivotal phase III clinical trials in castration resistant prostate cancer (CRPC) patients. Gal disrupts androgen receptor (AR) signaling via inhibition of CYP17, AR antagonism and AR degradation. Resistance to current therapy is attributed to up-regulation of full-length AR (fAR), splice variants AR (AR-Vs) and AR mutations. The effects of gal and VNPT55 were analyzed on f-AR and AR-Vs (AR-V7/AR^v567es^) in LNCaP, CWR22Rv1 and DU145 (transfected with AR-Vs) human PC cells *in vitro* and CRPC tumor xenografts. Galeterone/VNPT55 decreased fAR/AR-V7 mRNA levels and implicates Mdm2/CHIP enhanced ubiquitination of posttranslational modified receptors, targeting them for proteasomal degradation. Gal and VNPT55 also induced significant apoptosis in PC cells via increased Bax/Bcl2 ratio, cytochrome-c release with concomitant cleavage of caspase 3 and PARP. More importantly, gal and VNPT55 exhibited strong *in vivo* anti-CRPC activities, with no apparent host toxicities. This study demonstrate that gal and VNPT55 utilize cell-based mechanisms to deplete both fAR and AR-Vs. Importantly, the preclinical activity profiles, including profound apoptotic induction and inhibition of CRPC xenografts suggest that these agents offer considerable promise as new therapeutics for patients with CRPC and those resistant to current therapy.

## INTRODUCTION

The recurrence of prostate cancer (PC) with metastases following androgen deprivation therapy (ADT) is a major concern since it is the primary cause of death in patients initially diagnosed with localized PC [1–3]. This more aggressive stage of the disease is referred to as castration-resistant prostate cancer (CRPC). Since 2010, the US Food and Drug Administration (FDA) has approved five new agents, including, docetaxel plus prednisone, cabazitaxel, abiraterone acetate, sipuleucel-T and enzalutamide for the treatment of patients with CRPC [4, 5]. Despite these current therapeutic options, CRPC still presents a great clinical challenge due to inevitable development of drug resistance [5–10].

Resistance to current therapy has implicated ligand independent signaling and gene amplification of AR, mutations causing promiscuity [11] and emergence of splice variants of the AR that lacks the C-terminal domain (AR-Vs) [12]. Indeed, the lack of ligand binding domain (LBD) in AR-Vs is a potential cause of resistance to drugs that target AR via LBD [6–10, 13, 14]. The well-characterized AR-V7 (a.k.a., AR3) reportedly increases transcription of AR target genes in PC cells [13, 15–18].

It has been suggested that drugs that target the N-terminal domain (NTD) or regions other than the LBD of AR should be more potent in antagonizing both fAR and AR-Vs signaling [19]. However, we envision that agents that cause depletion of both fAR and AR-Vs (i.e., androgen receptor degrading agents, ARDAs) are most likely to have a major impact on hormone-sensitive prostate cancer (HSPC) and CRPC therapy [20, 21].

In our effort to discover potent and specific inhibitors of 17α-hydroxylase/17, 20-lyase (CYP17), the key enzyme which catalyzes the biosynthesis of androgens from progestins, 3β-(hydroxy)-17-(1*H*-benzimidazole-1-yl)androsta-5, 16-diene (galeterone or TOK-001, formerly called VN/124-1) was identified as a selective development candidate which modulates the androgen receptor (AR) signaling pathway at multiple points (reviewed in [20]). Using several *in vitro* and *in vivo* human prostate cancer models, we have established that galeterone (gal) inhibits CYP17, antagonizes fAR, and degrades both fAR and AR-Vs *in vitro*; and also reduces the expression of these proteins in tumor xenografts [22–30]. We also showed that gal inhibits AR positive and negative prostate cancer cells suggesting involvement of additional targets [22].

Two other groups have reported independent *in vitro* studies on the effects of gal in a variety of human prostate cancer cell lines. The report by Stein and colleagues concluded that gal and abiraterone down-regulated AR signaling via identical multiple mechanisms [31], while that by Balk and colleagues reported that gal prevents AR binding to chromatin and enhances degradation of mutant AR [32]. They even suggested that gal will function similar to enzalutamide in CRPC [32]. Some of the results in these two studies are in contrast to our many studies with gal in several *in vitro* models and recapitulated *in vivo* and in the clinic (reviewed in [20]; *vide infra*).

In recent studies, Tokai reported that gal suppressed castration-resistant and enzalutamide-resistant prostate cancer growth *in vitro* and also blocked nuclear translocation and decreased AR dependent genes (PSA, TMPRSS2, and Nkx3.1) [33]. Furthermore, recent clinical data show that administration of gal to four distinct CRPC patient populations, including treatment-naïve, non-metastatic; treatment-naïve, metastatic, abiraterone-refractory and enzalutamide-refractory patients resulted in clinically meaningful PSA reductions and an acceptable safety profile [34, 35]. Additionally, following a recent report by our group that gal also strongly degrades AR-V7 [21], Tokai conducted a retrospective study of their phase 2 clinical data and reported positive clinical data in patients with AR C-terminal loss, showing PSA50 response in 6 of 7 (85.7% response) CRPC patients with AR C-terminal loss, suggesting that gal has activity in AR-Vs-expressing CRPC patients [34, 35]. This data is in contrast to a recent study where no AR-V7-positive patient had any appreciable clinical benefit from enzalutamide or abiraterone therapy [6], which clearly differentiates gal from these related aforementioned androgen/AR targeting drugs. Gal is scheduled to enter pivotal phase III clinical trials in the 2^nd^ quarter of 2015 in CRPC patients positive for AR-V7 [20].

Understanding the multiple effects and pathways affected by investigational agents in modulating AR is essential in enhancing the design and synthesis of more potent and efficacious potential new drug agents [20, 21]. In addition, this new knowledge would enable rational use and possible combinations with other clinically approved drugs.

We report for the first time that posttranslational modulation of fAR and AR-V7 by gal and its 3β-carbamate analog, VNPT55 in LNCaP and CWR22Rv1 involves enhanced ubiquitination of these receptors. Our results implicate E3 ligases, Mdm2 and CHIP (C-terminus of Hsp70-interacting protein) in gal-induced AR/AR-V7 degradation. Interestingly AR^v567es^, which enhances fAR transcriptional activity [13, 36, 37] in metastatic CRPC is also degraded by gal and VNPT55. We also show for the first time that gal and its analog induce profound apoptosis in HSPC and CRPC cell lines. Importantly, gal and VNPT55 show robust anti-tumor efficacy in CRPC xenografts with significant depletion of AR/AR-V7 and a high Bax/Bcl2 ratio *in vivo*, with no apparent host toxicity. This study also highlights the effects or lack thereof of gal on non-target nuclear receptors and AR in normal prostate cells. To our knowledge, gal is currently the only known agent in clinical trials for prostate cancer that targets and depletes both the fAR and AR-Vs protein levels. A preliminary account of part of this work has recently been reported [38].

## RESULTS

### Effects of gal and VNPT55 on full length AR and splice variant AR-Vs

Recent efforts in our lab are focused on designing and synthesizing more potent analogs of gal (Supplementary Figure 1A) to target and deplete fAR/AR-V7 and inhibit the growth of prostate cancer cells [21]. Our initial attempt to compare gal and its new lead analog was determined using cell viability assays in LNCaP and CWR22Rv1 cells. VNPT55 exhibited stronger growth inhibitory activity (GI_50_ = 0.87 and 2.45 μM *vs*. LNCaP and CWR22Rv1, respectively) compared to gal (GI_50_ = 3.35 and 4.46 μM *vs*. LNCaP and CWR22Rv1, respectively).

**Figure 1:**
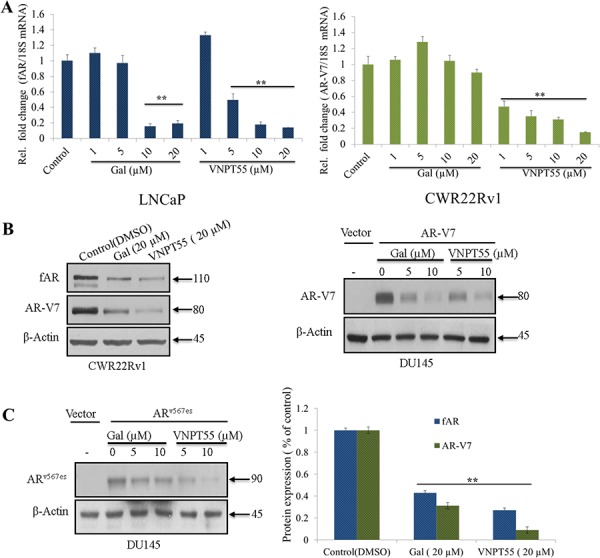

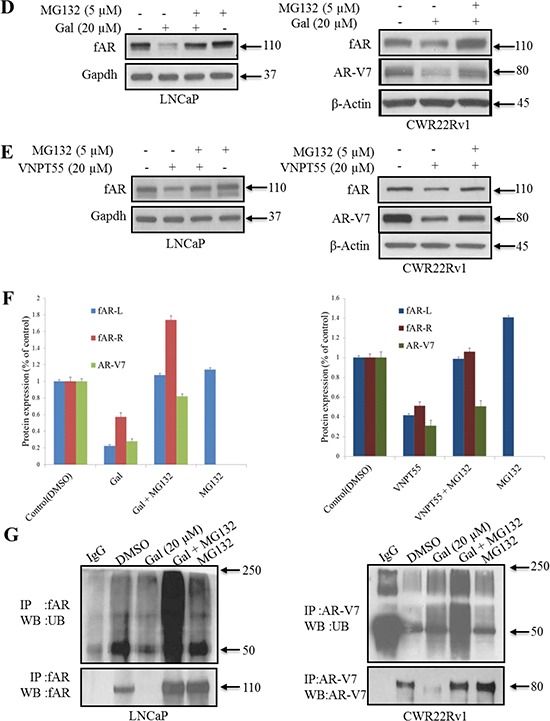
Effects of gal and VNPT55 on AR/AR-V7 mRNA and protein expression **A.** LNCaP cells treated with increasing concentrations of gal and VNPT55 (1, 5, 10, 20 μM) for 24 h and mRNA collected, qRT-PCR analysis of fAR mRNA; *p < 0.01, **p < 0.001. CWR22Rv1 cells were treated with increasing concentrations of gal and VNPT55 (1, 5, 10, 20 μM) for 24 h and mRNA collected, qRT-PCR of analysis of AR-V7 mRNA levels. **B.** (left panel) Effects of gal and VNPT55 on fAR/AR-V7 in CWR22Rv1 cells, **B.** (right panel) DU145 cells transfected with 0.5 μg of AR-V7 expression plasmid for 16 h and Cells subsequently treated with gal and VNPT55 (5 and 10 μM) for 24 h. **C.** (left panel), DU145 cells were transfected with AR^v567es^ expression plasmid as in B and treated with gal and VNPT55 (5 and 10 μM) for 24 h, **C.** (right panel) densitometry for three (3) replicates of experiments done in B (CWR22Rv1 Cells) **p < 0.001. **D.** (left panel) LNCaP and (right panel) CWR22Rv1 cells treated with gal at 20 μM for 16 h, thereafter, MG132 (5 μM) was added for an extra 8 h. Immunoblot analysis was carried out on fAR and AR-V7 protein expression. **E.** (left panels) LNCaP and (right panels) CWR22Rv1 were treated as in D with VNPT55. **F.** Densitometric analysis of D and E (fAR-L = full length AR in LNCaP cells, fAR-R = full length AR in CWR22Rv1 cells). **G.** LNCaP (left panel) and CWR22Rv1 (right panel) cells were treated as in D and E and 1 mg of total cell lysate used in immunoprecipitation and probed with ubiquitin antibodies.

Based on the recent report that gal decreases fAR mRNA in LNCaP cells [31], we compared the effects of gal and VNPT55 on fAR/AR-V7 mRNA levels. Both gal and VNPT55 decreased fAR mRNA levels in LNCaP cells (Figure 1A, left panel). Interestingly, although gal had no effect on AR-V7 mRNA, VNPT55 significantly decreased AR-V7 mRNA even at 1 μmol/L (Figure 1A, right panel) in CWR22Rv1 cells. It is interesting that minor modification to the parent compound increases the analog’s potency and efficacy in modulating fAR/AR-V7 at both transcriptional and posttranslational levels. To establish and compare the efficacies of gal/VNPPT55 on AR-Vs, we analyzed the potency of gal and VNPT55 in depleting protein levels of fAR/AR-V7 in CWR22Rv1 cells (Figure 1B, left panel). In agreement with our earlier report [21], the efficacy of VNPT55 on cell viability and AR-induced depletion appears to be superior to that of gal.

It is well documented that CWR22Rv1 cells express a number of AR-Vs [16, 39]. Thus, to eliminate the possibility of AR-V7 antibody cross-reacting with other AR-Vs and to specifically determine its effects on particular AR-Vs, DU145 cells were transfected with AR-V7 expression plasmid and treated with gal or VNPT55. At concentrations of 5 and 10 μM, each compound significantly depleted AR-V7 (Figure 1B, right panel). Transfection of AR^v567es^ (AR splice variant expressed in CRPC, [13]) and subsequent treatment with gal/VNPT55 yielded similar results (Figure 1C, left panel). Densitometry analysis of the efficacy of gal and VNPT55 on AR/AR-V7 protein levels shows a strong decrease of AR/AR-V7 to 0.4/0.3 after gal treatment and a more profound decrease after VNPT55 treatment to 0.3/0.09 of AR/AR-V7, respectively (Figure 1C, right panel).

The proteasomal pathway has been described as one of the major mechanisms regulating AR turnover [40–43]. To determine the involvement of the 26S proteasome in gal- or VNPT55-enhanced depletion of AR/AR-V7 protein, we co-treated cells with gal or VNPT55 and a proteasome inhibitor, MG132, in LNCaP and CWR22Rv1 cells. As shown in Figure 1D, E and 1F, proteasomal inhibition significantly rescued gal- and VNPT55 induced fAR/AR-V7 degradation in both LNCaP and CWR22Rv1 cells. Our previously published report implicated intracellular calcium ion ([Ca^2+^]_i_) release and induction of endoplasmic reticulum stress response (ERSR) in PC-3 PC cells as a possible mechanism of action of gal [22]. Because other studies have established Ca^2+^-dependent, calpain-mediated breakdown of f-AR in human PC cells [44–47], we consider it important to determine whether gal’s depletion of AR may implicate calpains. Indeed, although inhibition of calpains with calpeptin (Cal) did not entirely rescue gal/VNPT55-induced AR degradation (Supplementary Figure 1B, C and D), it indicates to a minor extent (~20%), that the possible release of Ca^2+^ induced by gal treatment enhanced AR depletion.

Since ubiquitination precedes 26S proteasomal protein degradation [48], we examined whether gal enhanced AR and AR-V7 ubiquitination. Gal treatment in the presence of MG132 significantly increased AR ubiquitination compared to MG132 alone (Figure 1G, left panel) in LNCaP cells. In CWR22Rv1 cells (Figure 1G, right panel), immunoprecipitated AR-V7 in the presence of gal and MG132 interestingly also shows an increase in AR-V7 ubiquitination.

### Implication of Akt and Mdm2 phosphorylations in gal/VNPT55-induced AR degradation

**Figure 2:**
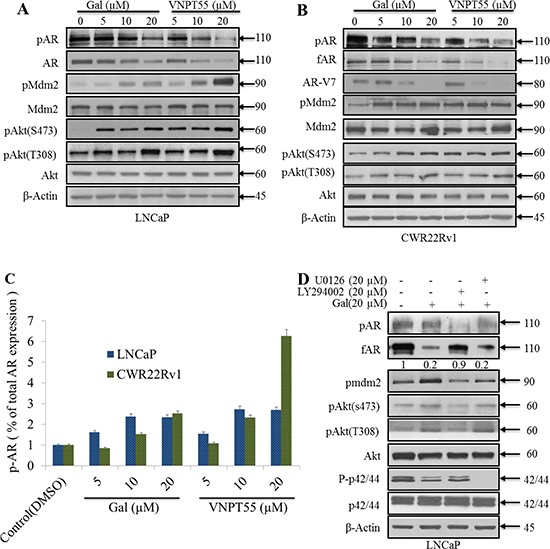

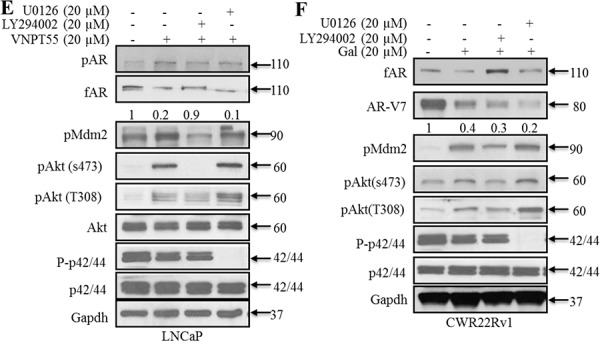
Significance of Akt and Mdm2 phosphorylation in gal/VNPT55 induced fAR/AR-V7 degradation **A.** and **B.** LNCaP and CWR22Rv1 cells, serum starved for 12 h were treated with increasing concentrations (5, 10 and 20 μM) of gal or VNPT55 for 24 h. **C.** Densitometry was done for phosphorylated levels of AR, normalizing p-AR to the total AR expression after gal and VNPT55 treatment. **D.** and **E.** LNCaP cells were serum starved for 12 h and pre-treated with 20 μM LY294002 (PI3k inhibitor) or 20 μM U0126 (MAPK inhibitor) for 1 h and then treated with gal/VNPT55 (20 μM). **F.** CWR22Rv1 cells were serum starved in phenol red free media supplemented with charcoal dextran FBS for 12 h and treated with gal (20 μM) for 24 h after pretreating with LY294002 (20 μM) or U0126 (20 μM) for 1 h. Samples were immunoblotted for full length AR (fAR), AR-V7, phospho Akt, phospho Mdm2 and phospho p42/44, using Gapdh as loading control.

PI3k activation induces phosphorylation of Akt (at Ser473 and Thr308), Mdm2 (at Ser166) and AR (at Ser210/213 and Ser790) this posttranslational modification can serve as a signal for AR ubiquitylation and subsequent degradation [40, 42, 43, 49]. We next examined the effects of gal and VNPT55 on Akt and Mdm2 phosphorylations. As shown in Figure 2A and 2B, treatment of LNCaP or CWR22Rv1 cells with gal or VNPT55, resulted in increased phosphorylations of Akt at Thr308 and Ser473. Mdm2 phosphorylation at Ser166 also increased significantly. It is important to note that, although phosphorylated levels of AR seems to decrease (see band intensities), the relative levels of phosphorylated AR to total AR show an increase in AR phosphorylation, both in LNCaP and CWR22Rv1 cells (Figure 2C, densitometry). Total AR levels decrease due to depletion effects of gal and VNPT55, so in order to quantify phosphorylated AR levels we calculated the p-AR/AR ratio (Figure 2C).

We then investigated the significance of Akt/Mdm2 phosphorylation in gal/VNPT55-induced AR/AR-V7 depletion. Pharmacological inhibition of PI3k (LY294002, 20 μM) in LNCaP cells co-treating with gal/VNPT55 suppressed agent-induced AR degradation significantly (~90%) (Figure 2D and 2E). In CWR22Rv1 (Figure 2F) cells, we observed that rescue of gal-induced fAR degradation was similar to the trend in LNCaP cells in Figure 2D. In contrast, AR-V7 depletion was not rescued, but was further depleted compared to gal alone treatment. It is well-established that the lack of LBD and thus loss of N-C interaction changes AR-V7 dynamics and interacting proteins [50, 51]. It is reasonable to suggest that this phenomenon may contribute to the effects seen when gal is combined with LY294002.

The PI3k-Akt and the mitogen activated protein kinase (MAPK) pathways both crosstalk with the AR and induce its phosphorylation at different serine and threonine residues [40, 52, 53]. To rule out any possible involvement of MAPK pathway in gal/VNPT55-induced AR/AR-V7 modulation, U0126 (20 μmol/L), a MAPK inhibitor, was co-incubated with gal in both LNCaP and CWR22Rv1 cells. As shown in Figure 2D, E and 2F, the ability of gal to degrade fAR and AR-V7 were not blocked in the presence of U0126.

It has been reported that both LY294002 and UO126 exhibits off-target effects and decreases AR protein expression [54–57]. In agreement with previous reports, we observed that U0126 cause suppression of AR (Supplementary Figure 2A). In addition, we show that U0126 decreased AR-V7 expression in CWR22Rv1 cells (Supplementary Figure 2B). In contrast to the effects of LY294002 or U0126 on fAR degradation, the combination of these inhibitors with gal cause enhanced AR-V7 degradation (Figure 2F). It is possible that the loss in N-C interaction in AR-V7 and as a consequence, the loss in c-terminal phosphorylation sites needed to enhance AR-V7 stabilization and/or possible off-target effects of the inhibitors may be potentiated by gal. Collective, these data suggest that unlike MAPK kinase, PI3k/Akt kinase plays a significant role in gal-induced fAR degradation.

### Galeterone enhances fAR/AR-V7-Mdm2/CHIP interaction

**Figure 3:**
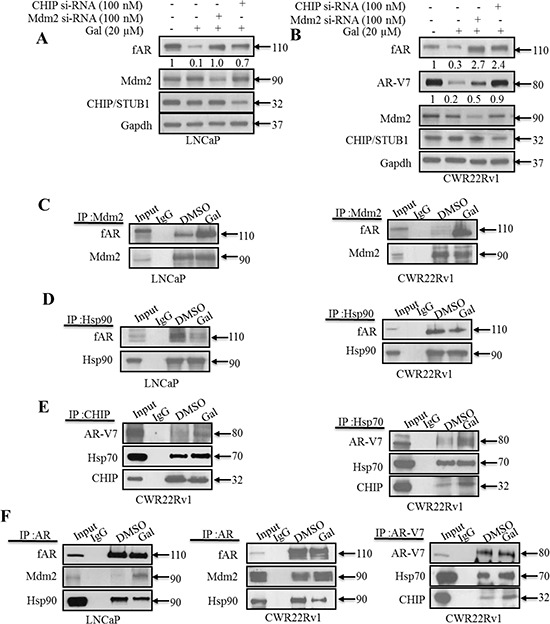
Gal enhanced AR/AR-V7 degradation implicates E3 ligase Mdm2 and CHIP **A.** LNCaP cells seeded at 60% confluency were transfected with Mdm2 or CHIP siRNAs (100 nM each), with scrambled siRNA as control. Cells were then exposed to 20 μM gal for 24 h. **B.** CWR22Rv1 cells were subjected to similar transfection and treatment conditions as in A, separated proteins were probed for fAR, AR-V7, Mdm2 CHIP. **C.** LNCaP (left panel) and CWR22Rv1 (right panel) cells were serum starved for 12 h and treated with 10 μM of gal for 14 h; 1 mg of total cell lysate was subjected to immunoprecipitation with Mdm2 rabbit polyclonal antibodies. **D.** LNCaP (left panel) and CWR22Rv1 (right panel) cells were treated as in C and subjected to immunoprecipitation analysis with Hsp90 rabbit polyclonal antibodies. Samples were immunoblotted for Mdm2, Hsp90 and AR with mouse monoclonal antibodies. **E.** (left and right panels) 1 mg of total cell lysates from CWR22Rv1 cells treated with 10 μM of gal for 11 h was subjected to immunoprecipitation with CHIP and Hsp70 polyclonal antibodies, respectively, to analyze Hsp70-CHIP-AR-V7 interaction. **F.** Co-immunoprecipitation assays in LNCaP and CWR22Rv1 cells with AR (N20) polyclonal and AR-V7 mouse monoclonal antibodies were carried out with 1 mg cell lysates after treating with gal (10 μM) for 14 h (LNCaP cells) and 11 h (CWR22Rv1 cells). Membranes were probed for AR, AR-V7, Mdm2, Hsp90 and Hsp70 mouse monoclonal antibodies.

Mdm2 and CHIP are two of the well-studied and established E3 ligases involved in AR proteasomal degradation [51, 58, 59]. To determine their involvement in gal-induced fAR/ARV7 degradation, Mdm2 or CHIP were silenced using small interfering RNA (siRNA) in LNCaP or CWR22Rv1 cells followed by treatments with gal for 24 h. As shown in Figure 3A, although both Mdm2 and CHIP knockdown inhibited fAR degradation, the effect was more significant with Mdm2 knockdown in LNCaP cells. Conversely, in CWR22Rv1 cells, silencing of CHIP E3 ligase exhibited a higher rescue effect on gal-induced degradation of AR-V7 (Figure 3B). We also knockdown Mdm2 or CHIP and in the absence of gal, there was no significant effect on fAR or AR-V7 (Supplementary Figure 2C and D).

Mdm2 has previously been reported to interact and enhance AR ubiquitination preceding its degradation via the 26S proteasome [43]. From our siRNA knockdown experiments (Figure 3A), we hypothesized that gal enhanced AR-Mdm2 and AR-V7-CHIP interactions, thereby increasing the receptors’ ubiquitination and subsequent degradation. To ensure immunoprecipitating equal amount of proteins, specifically fAR/AR-V7, in both control and treated groups, cells were treated for either 11 or 14 h with gal at 10 μM and total cell lysates analyzed prior to immunoprecipitation experiments (Supplementary Figure 2E and F). Input lanes are from control (DMSO) treated cell lysates to confirm molecular weights of immunoprecipitated proteins. Treatments with gal greatly enhanced fAR-Mdm2 interaction in both LNCaP and CWR22Rv1 cells (Figure 3C). Heat shock protein 90 (Hsp90) is known to interact with AR in a heteromeric complex which enhances stability of the non-ligand bound receptor [40, 42, 51, 60]. Hsp90 inhibitors such as 17-AAG induce AR-Hsp90 dissociation and enhance AR degradation [61]. We determined the effects of gal on Hsp90-fAR interaction. Interestingly, we found that in both LNCaP and CWR22Rv1 cells, gal treatment significantly reduced this interaction (Figure 3D).

Results from immunoprecipitation assays were consistent with our siRNA experiments, in that the CHIP E3 ligase is more significant in gal-induced AR-V7 ubiquitylation and degradation. Gal enhanced association between CHIP and AR-V7 (Figure 3E, left panel). A recent report on the possibility of Hsp70-AR-V7 interaction [60], prompted us to investigate the effects of gal on this interaction if any. Indeed, we found that gal enhanced Hsp70-AR-V7 interaction (Figure 3E, right panel), suggesting Hsp70 involvement in the formation of a degradation complex. The Co-IP with AR confirmed increased interactions in LNCaP and CWR22Rv1 cells, respectively, between fAR and Mdm2 and a loss in interaction between fAR and Hsp90 (Figure 3F, left and middle panels). As shown in Figure 3F right panel, we immunoprecipitated AR-V7 from CWR22Rv1 cell lysates and observed an increase in interacting proteins CHIP and Hsp70. These data suggest that, changes observed in Hsp90-AR and Hsp70-AR-V7 interactions after gal treatment possibly rendered the receptors more susceptible to proteasomal degradation, in agreement with documented reports of small molecule-induced fAR degradation via the ubiquitin/proteasome-mediated proteolysis pathway [40, 42, 51, 60].

Taken together, these results strongly suggest that gal/VNPT55-induced fAR/AR-V7 protein degradation is mediated predominantly through the ubiquitin/proteasome pathway that involves Mdm2 and CHIP E3 ligases.

### Gal and VNPT55 induce significant apoptosis via cytochrome c release and caspase-dependent PARP cleavage

**Figure 4:**
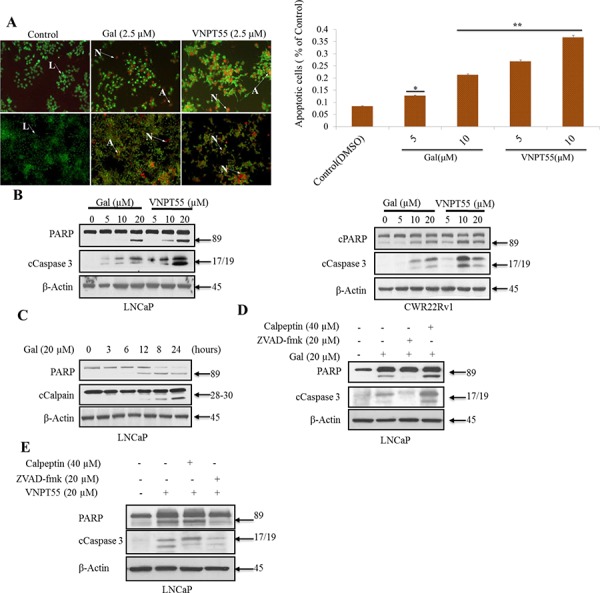

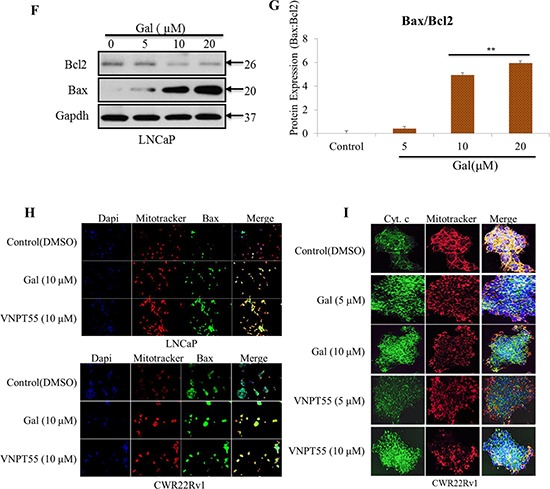
Gal and VNPT55 induce apoptosis in LNCaP and CWR22Rv1 cells **A.** (left panel) Apoptotic induction was analyzed with the acridine orange/ethidium bromide (AO/EB) staining assay in LNCaP (top panel) and CWR22Rv1 (bottom panel) cells. Indicated cells lines were treated with 2.5 μM of gal or VNPT55 for 72 h. Cells were washed 1X gently with warm PBS and incubated with a 1:2 ratio of ethidium bromide: acridine orange in PBS for 30 minutes. Cells were washed 1X again and images taken with a fluorescence microscope Nikon TE2000. Live, apoptotic and necrotic cells stain green, orange and red respectively. Arrows next to L indicate live cells; arrows pointing to A indicate apoptotic cells; and arrows pointing N indicate necrotic cells. **A.** (right panel), CWR22Rv1 cells treated with gal and VNPT55 at 5 and 10 μM after 24 h were collected and washed 2X with PBS. Cells were then stained with annexin-v and PI. FACS analysis was performed for apoptotic cells after gal and VNPT55 treatment at 5 and 10 μM (*p < 0.01, **p < 0.001). **B.** LNCaP (left panel) and CWR22Rv1 (right panel), cells were serum starved in phenol-red free media supplemented with charcoal dextran cFBS for 12 h and treated with increasing doses of gal or VNPT55 (5, 10 and 20 μM) for 24 h. **C.** LNCaP cells were treated with gal at 20 μM for different time points (0, 3, 6, 12, 18, 24 h). **D.** & **E.** LNCaP cells were exposed to gal or VNPT55 at 20 μM in combination with calpeptin (40 μM) or ZVAD-fmk (20 μM) for 24 h. **F.** LNCaP cells serum starved for 12 h were treated with gal (5, 10 and 20 μM) for 24 h and immunoblotted for Bax and Bcl2. **G.** Densitometry analysis of Bax/Bcl2 ratio show an increase with increase in gal concentration (**p < 0.001). **H.** (top panel) Bax co-localizes with mitochondria and enhances mitochondrial permeability. LNCaP cells were treated with 10 μM galeterone or VNPT55 for 24 hours. Cells were then stained with mitotracker (RED CMXRos) and Bax antibody. (bottom panel) CWR22Rv1 cells were treated as in LNCaP cells and subsequently stained with mitotracker and Bax antibody. Images were taken and merged. Areas of co-localization are yellow. **I.** CWR22Rv1 cells were seeded in an 8-chamber slide and treated with gal or VNPT55 (5 and 10 μM) for 24 h. Cells were then incubated with 125 nM of mitotracker at 37°C for 15 minutes after which cells were fixed with 3.7% paraformaldehyde and stained with cytochrome c at 4°C for 12 h.

Considering the strong impact that gal or VNPT55 have in promoting the degradation of AR/AR-Vs proteins, and thus, their preclinical anti-prostate cancer activities and their efficacy in the clinic (*vide supra*), another important focus in this study was also to determine whether these compounds induce apoptosis in PC cells. Apoptotic induction was initially evaluated by the acridine orange/ethidium bromide (AO/EB) dual staining assay [60]. Treatment of LNCaP or CWR22Rv1 with gal or VNPT55 (2.5 μM each) for 72 h induced apoptosis as assessed by loss of cell membrane integrity and nuclear fragmentation (cells stained yellow/reddish orange) (Figure 4A, left panel). Cells undergoing apoptosis loose membrane integrity enabling ethidium bromide to penetrate cells, whereas viable cells are only permeable to acridine orange which stains nuclear green [62–64].

Furthermore, we also assessed gal/VNPT55 apoptotic induction by flow cytometry. CWR22Rv1 cells treated for 24 h with gal and VNPT55 (5 and 10 μM) were stained with annexin-v and propidium iodide, following manufacturers protocol. Interestingly, we observed that at 5 μM, gal and VNPT55 significantly induced apoptosis in CWR22Rv1 cells (Figure 4A, right panel). Further analysis on PARP cleavage, a well-established signature of apoptosis [65], shows a dose-dependent effect on activated caspase 3 and cleaved PARP in both LNCaP (Figure 4B, left panel) and CWR22Rv1 cells (Figure 4B, right panel).

As stated earlier, we previously reported that gal modulates intracellular Ca^2+^ levels and endoplasmic reticulum stress response (ERSR) [22]. Intracellular Ca^2+^ have previously been implicated in the modulation of calpains that also induce apoptosis by cleaving a variety of pro-apoptotic proteins such as calpain 1 which cleaves Bid to enhance cytochrome c release [66]. Thus, we directed our efforts to determine whether gal-induced apoptosis was as a result of activated calpains, and/or solely due to other well-established pathways. Initial attempts with time-dependent analysis of gal on PARP cleavage showed this to coincide with calpain activation at 12 hours post gal treatment (Figure 4C). To assess whether gal/VNPT55-induced PARP cleavage is a consequence of caspase activation via other mechanisms or due to calpain activation, LNCaP cells were treated with gal in the presence or absence of the caspase inhibitor, ZVAD-fmk, or the calpain inhibitor, calpeptin. Data presented in Figure 4D and 4E shows that both gal and VNPT55-induced PARP cleavage was predominantly not via calpain activation.

Since calpain inhibition did not significantly inhibit caspase 3 and PARP cleavage, we focused on Bax, a proapoptotic protein which could enhance mitochondrial permeability [63, 67, 68]. LNCaP cells treated with increasing doses of gal resulted in significant and remarkable enhancement of the pro-apoptotic protein, Bax and a modest decrease in anti-apoptotic Bcl2 (Figure 4F), leading to profound increase of the Bax/Bcl2 ratio (Figure 4G).

An increase in Bax and its translocation to the mitochondria has been implicated in cytochrome c release into the cytosol [69–71], and it has also been shown that Bax co-localization with mitochondria results in Bax protein pore formation to enhance the release of mitochondrial intermembrane protein [69, 72, 73]. We therefore stained for Bax-mitochondria co-localization in both LNCaP and CWR22Rv1 cells after 24 h treatments with gal or VNPT55. As shown in Figure 4H gal/VNPT55 treatments cause Bax co-localization with mitochondria (yellow stains). To show cytochrome c release as a result of Bax translocation and pore formation in the mitochondria, we stained CWR22Rv1 cells with mitotracker and cytochrome c after a 24 h gal/VNPT55 treatment. As illustrated in Figure 4I, we see a significant release of cytochrome c, as indicated by co-localization of cytochrome c (green) and mitotracker (red) in control (DMSO) contrary to treated cells.

In summary, gal and VNPT55 induce profound apoptosis in LNCaP and CWR22Rv1 cells. Importantly, these promising and robust data differ from previous reports which suggest that current PC drugs that block AR transactivation do not cause significant apoptosis, which is believed, in part, to be the basis of their failure in the clinic [19].

### Effects of Gal and VNPT55 on nuclear receptors in LNCaP cells and AR in PWR-1E and WPMY-1 prostate cells

**Figure 5:**
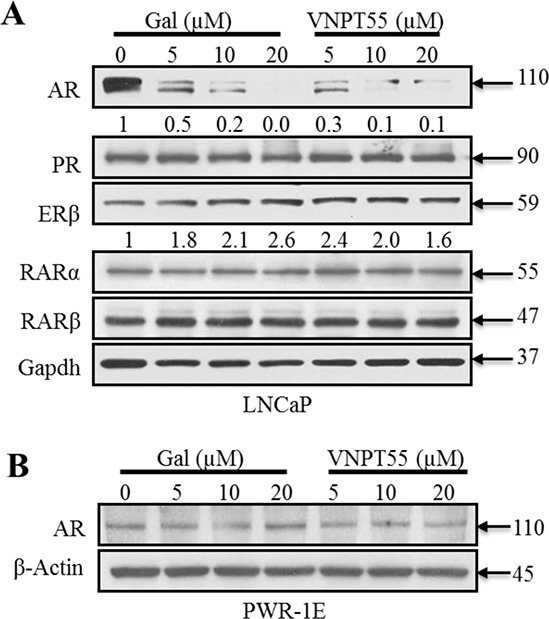
Effects of gal/VNPT55 on AR in epithelial prostate cells and nuclear receptors in LNCaP cells **A.** 12 h serum starved LNCaP cells were treated with gal (5, 10, 20 μM) or VNPT55 (5, 10, 20 μM) for 24 h, using DMSO as control. **B.** Immortalized untransformed prostate epithelial cells (PWR-1E cells) were incubated in phenol red free charcoal dextran FBS for 12 h and treated with gal or VNPT55 at 5, 10 and 20 μM for an additional 24 h.

To demonstrate the specificity of gal/VNPT55 and evaluate any off-target effects, we assessed the effect of gal or VNPT55 on the levels of related nuclear receptor proteins in LNCaP cells, including, progesterone receptor (PR), estrogen receptor beta (ERβ) and retinoic acid receptors (RAR-α and -β), which are known to be regulated by the proteasome-mediated pathway [74]. As shown in Figure 5A, whereas gal/VNPT55 caused profound depletion of AR, no change in PR, RARα and RARβ protein levels were detected. In addition, and as expected, there was a modest up-regulation of ERβ (a PC tumor suppressor) [75], as a consequence of AR depletion [76]. Interestingly, gal had no significant effect on AR expression in immortalized untransformed PWR-1E (epithelial) prostate cells (Figure 5B). These data suggest that gal and VNPT55 exhibit cell selective induction of AR degradation.

### Galeterone and VNPT55 suppress CWR22Rv1 xenograft tumor growth and AR/AR-V7 in castrated SCID mice

**Figure 6:**
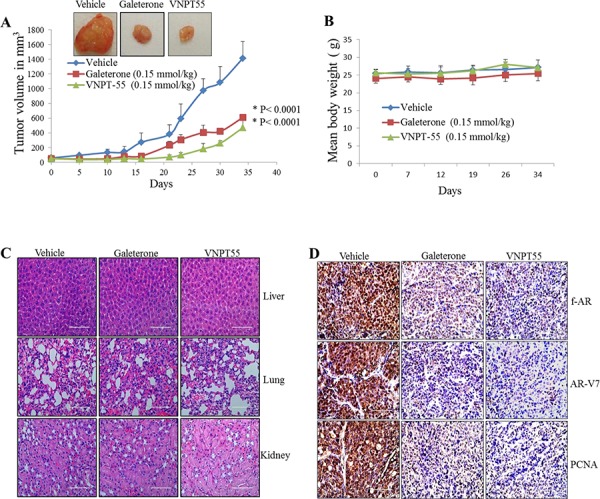

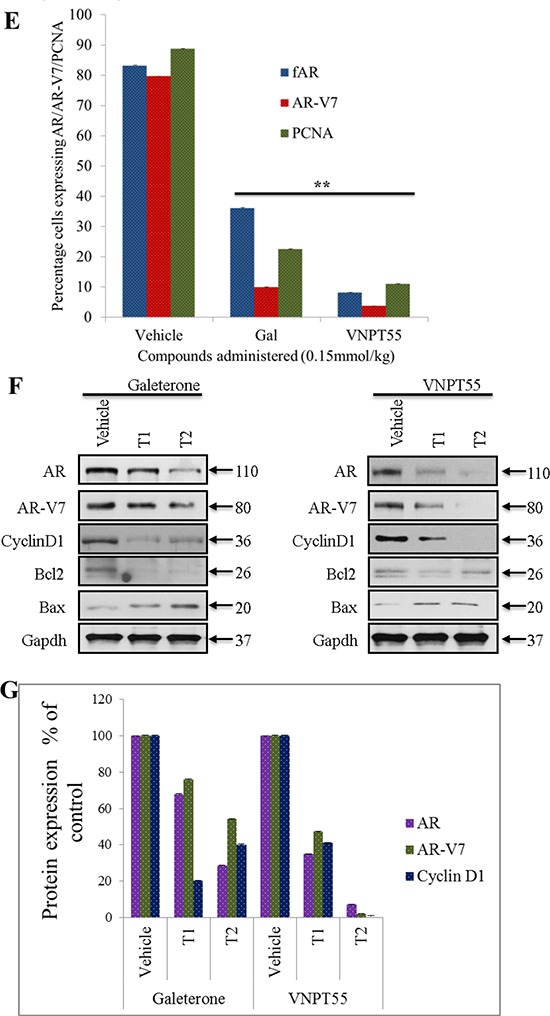
Antitumor activity of galeterone and VNPT55 in a CWR22Rv1 xenograft **A.** Representative tumors from the 2 groups. Effect of gal and VNPT55 was evaluated in castrated CWR22Rv1 xenograft-bearing mice. Mice (*n* = 5) were administered with gal (0.15 mmol/kg/twice daily) and VNPT55 (0.15 mmol/kg/twice daily), by intraperitoneal injection, 5 days per week for 34 days. Tumors were measured twice a week. **B.** Mean body weights of mice were weighed once a week for the duration of the study. **C.** Hematoxylin and eosin staining of normal organs, formalin fixed and paraffin embedded tissues to show *in vivo* and off-target toxicity or not of compounds. **D.** Representative images of full length AR, AR-3 and PCNA immunostaining in vehicle and treated groups. **E.** ImageJ was used to quantify Immunohistochemical staining in D. **E.** ImageJ was used to quantify Immunohistochemical staining in D. **F.** Xenograft tumor tissues were harvested and analyzed by western blotting. Effects on fAR, AR-V7, cyclin D1, Bcl2 and Bax were analyzed in both galeterone and VNPT55 groups compared to controls. **G.** Densitometry analysis of protein expression from western blot analysis was plotted to quantify the effects seen *in vivo*.

Gal is currently in clinical development for the treatment of CRPC. Because splice variant AR-V7 plays a significant role in castration resistance, we further evaluated the efficacy of gal and VNPT55 in CRPC AR-V7 positive CWR22Rv1 xenografts. Cells inoculated on flanks of mice after castration grew into sizeable tumors even with complete ADT (Figure 6A). Castrated male SCID mice bearing CWR22Rv1 tumors were treated with vehicle, gal or VNPT55 for 34 days as described in Materials and Methods. As shown in Figure 6A, gal and VNPT55 significantly inhibited CWR22Rv1 tumor growth by 60% (*p* < 0.0001 *vs*. vehicle) and 70% (*p* < 0.0001 *vs*. vehicle), respectively. In addition, no host toxicity was observed as monitored by changes in body weight throughout the study (Figure 6B). The H & E staining of liver, lung and kidney in the treated groups did not show any gross organ abnormalities on histological examinations (Figure 6C).

To further validate the anti-prostate cancer activities of gal and VNPT55 seen *in vitro*; we evaluated the expression levels of f-AR, AR-V7 and cell cycle and apoptosis-related proteins *in vivo* using representative tumor samples. Immunohistochemistry analysis with anti-fAR and AR-V7 antibody on tumors showed that gal and VNPT55, significantly reduced intensities and expressions of fAR and AR-V7 in treated samples (Figure 6D) In addition, a significant decrease in the expression of proliferating cell nuclear antigen (PCNA) was observed in gal and VNPT55 treated tumors, suggesting the inhibitory effects on cell cycle *in vivo* (Figure 6D). Immunohistochemical stain quantification of Figure 6D shows the significant decrease in protein expression *in vivo* (Figure 6E). Western blot analysis further confirmed that gal and VNPT55 degrade both fAR and AR-V7 in the tumors. As shown in Figure 6F, both agents caused significant depletion of cyclin D1 and Bcl2 and an increase in Bax expression. Densitometry analysis of fAR, AR-V7 and cyclin D1 protein expression in two representative tumors in the treatment groups and the vehicle treated group are represented in Figure 6G. Taken together, these data show that gal and VNPT55 suppress the growth of CRPC xenograft tumors in complete androgen-deprived conditions possibly via degradation of both fAR and its constitutively-active splice variant, AR-V7, and induction of apoptosis.

## DISCUSSION

**Figure 7:**
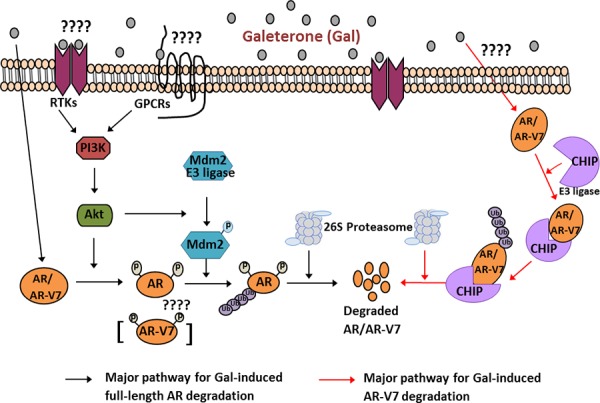
Schematic representation for proposed mechanisms of Gal on full length AR and splice variant AR-V7 degradation 1) Major pathway for gal-induced fAR degradation (left). Gal treatment of PC cells implicates PI3K/Akt pathway: Akt phosphorylates AR at Ser210. Active Akt also phosphorylates Mdm2, which enhances ubiquitination of AR. Ubiquitinated AR is degraded by the 26S proteasome. 2) Major pathway for gal-induced AR-V7 degradation (right)). Gal treatment of PC cells stimulates increased interaction between CHIP-AR-V7, which then enhances ubiquitination of AR-V7 and targets it to degradation via the 26S proteasome.

Androgen receptor pathway antagonism is one of the major strategies in prostate cancer therapy. Currently gal’s efficacy against HSPC and CRPC cancer cells is strongly related to its multiple effects. Additional inhibitory effects of gal on AR binding to chromatin were reported by Balk and colleagues [45]. Overexpression of fAR and AR-Vs is implicated in resistance to current approved therapy in HSPC and CRPC [11, 12]. Through the use of clinically relevant PC cell lines and pharmacologic inhibitors, we demonstrated that fAR and AR-V7 protein degradation by these two novel agents involves predominantly, the ubiquitin-proteasome pathway. The potential roles of Mdm2 and CHIP E3 ligases in gal-induced degradation of fAR and AR-V7, respectively, are summarized in a model in Figure 7.

Interestingly, the fact that fAR and AR-V7 mRNA levels and their protein levels following treatment of PC cells with gal or VNPT55 were significantly depleted, as well as AR^v567es^ protein levels, suggest that these agents could deplete a wide range of AR mutants or variants that are expressed in CRPC. AR is known to auto-regulate its transcription [76], and a decline of AR protein would be expected to cause an increase in AR transcription. However, the fact that we observe a decrease in AR mRNA and protein depletion suggests that gal and its analog could sustain a down-regulation of AR in PC patients. It is also important to note that resistance and proliferation abilities of PC cells due to activated pathways by non-genotropic activities of AR [78] will be eliminated resulting in an enhanced anti-cancer activity.

Further mechanistic analysis revealed that pharmacological inhibition of Akt phosphorylation suppressed gal-induced fAR degradation in contrast to the effects seen with AR-V7 degradation. Single agent treatment with LY294002 did not significantly affect AR-V7 protein expression (Supplementary Figure 2B), however the combination decreased its expression further, and this was the case with MAPK inhibitor also, which leads a potential future combination treatment studies to target AR-V7. From our data, Akt and Mdm2 phosphorylation was significantly implicated in fAR degradation in both LNCaP and CWR22Rv1 cells. Using targeted siRNA knockdown of Mdm2/CHIP and immunoprecipitation assays we demonstrated that, unlike fAR, gal induction of AR-V7 degradation significantly involved enhanced interaction with CHIP E3 ligase. The lack of C-terminal domain of the splice variant AR-V7 has been reported to change its dynamics and cofactors impacting protein-protein interactions [79]. It is possible that this truncation also changes protein-protein interactions induced by gal on the variant AR to favor CHIP E3 ligase mediated AR-V7 degradation. Further studies on AR-V7-CHIP interaction are needed to fully delineate the significant factors involved in AR-V7 modulation. A recent report suggests that AR phosphorylation impairments may favor CHIP E3 ligase-mediated fAR proteasomal degradation [80]. This may be the case with gal-induced AR-V7 degradation.

Gal induced dissociation of fAR from Hsp90 after treatment in LNCaP cells. Although there is sparse literature on AR-Vs and their interactions with co-activators/chaperones, Weigel and colleagues recently reported that unlike fAR, AR-V7 is resistant to inhibitors of the Hsp90-AR heterocomplex and suggested possible existence of Hsp70-AR-V7 complex [60]. Our data appear to be consistent with this report, because AR-V7 degradation was preceded by Hsp70 and CHIP association. Perhaps, what’s more interesting is that, although AR-Hsp90 disruptive inhibitors have no effect on AR-V7, we show that AR-V7 undergoes ubiquitination and proteasomal degradation and hence can be targeted via that pathway.

In addition to gal’s well-established triple mechanism of anti-AR activity [21, 30], we report for the first time that gal-induces robust apoptosis in PC cells *in vitro* and *in vivo*. Induction of caspase-3 and PARP cleavage is possibly one of the major reasons for the efficacy of gal and its analog in inhibiting PC cell growth. We note that the apoptosis-inducing effect of an agent is also dependent on the balance of anti-apoptotic and pro-apoptotic proteins (Bcl2 and Bax) [81]. Typically, the ratio of Bcl2 and Bax protein expression is used as an index of apoptosis [82]. We observed a remarkable up-regulation of Bax protein levels and a moderate decrease in the Bcl-2 protein levels, leading to an increase of the Bax/Bcl-2 ratio. This observation is important, as induction of robust apoptosis is crucial for inhibition of growth and progression of PC, and may be considered as a paramount predictive marker for forecasting the clinical therapeutic response to gal and its analogs. Depletion of Bcl2 may also contribute significantly to the anti-cancer efficacy of gal, as Bcl2 is known to cause chemo-resistance in prostate cancer [83].

To confirm that most of these effects seen on the various pathways were not as a result of increased utilized doses, we compared induction of apoptosis at different doses (2.5, 5, 10 and 20 μmol/L) over 24 and 72 h, using different complementary analytical tools. We also evaluated the effects on AR/AR-V7 at low concentrations with positive results (Supplementary Figure 1E and F). This suggests that the effects of gal and VNPT55 (at concentrations ranging from 1 to 20 μmol/L) on fAR/AR-V7 and apoptosis are not off target or toxic effects. The fact that gal and VNPT55 also induce calpain-dependent AR depletion led us to conclude that gal and VNPT55 induce well-established cell based mechanisms to inhibit cell proliferation.

Remarkably, gal and VNPT55 had no significant effect on the expression of fAR in immortalized untransformed prostate epithelial cells (PWR-1E). This is very significant as it gives an insight to the low toxic nature of the agent, as observed in the clinic [20, 34, 35], and the potential of a minimal effect on AR expressed in healthy non-cancerous tissues. This finding is consistent with the ability of agents to preferentially induce depletion of AR expression in PC tumors, without affecting AR expression in normal prostate cells, as recently reported by others [49, 84].

Perhaps the most significant piece of data from this study is the anti-tumor efficacy of gal and VNPT55 in fAR/AR-V7 positive CWR22Rv1 xenografts (a difficult-to-treat CRPC model). Gal/VNPT55 significantly inhibited tumor growth compared to vehicle treated groups, with no apparent host toxicity. Gal and VNPT55 also degraded both fAR, AR-V7 *in vivo* and also depleted cyclin D1 and Bcl2, but enhanced the level of Bax. In strong support of their mechanisms of anti-tumor activities, most effects seen *in vitro* were recapitulated *in vivo*.

One major mechanism of resistance in CRPC is the overexpression of AR and the emergence of splice variant ARs (AR-Vs). Recently approved PC drugs, i.e., abiraterone and enzalutamide have been reported to cause rapid induction and increased expression of AR-Vs in laboratory and clinical settings [6–10, 13, 14]. In addition, anti-androgens currently used for the treatment of PC do not cause significant apoptosis that may contribute to their failures in the clinic [19]. The present study shows significant gal-induced degradation of fAR, AR-V7 and AR^v567es^ that are implicated in all stages of PC progression and resistance settings [6–10, 13, 14]. Our *in vivo* CRPC xenograft data validated the effects observed *in vitro*. Overall, this study has high translational relevance because it provides strong preclinical validation of phase II results and the proposed pivotal phase III clinical trials of gal in PC patients with AR-Vs.

## MATERIALS AND METHODS

### Cell lines and reagents

CWR22Rv1, LNCaP and DU145 cells, and immortalized untransformed prostate epithelial cells (PWR-1E) were purchased from ATCC. PI3k inhibitor (LY294002), MAPK inhibitor (U0126), rabbit polyclonal antibodies against AR, pMdm2, β-actin, Gapdh, pAkt (S473), pAkt (T308), CHIP, PARP, caspase 3, pMdm2, p42/44, phospho p42/44, secondary antibodies, anti-mouse and anti-rabbit HRP were purchased from Cell Signaling. Cell culture reagents (FBS, RPMI, and DMEM) were from Invitrogen. Gal and VNPT55 were synthesized in our laboratory as previously reported [21, 25]. PhosphoAR (pAR) was purchased from Imgenex. AR-V7 (AR3) expression plasmid was obtained from Dr. Yun Qiu, University of Maryland, Baltimore. AR-V7 antibody was purchased from Precision Antibodies. Dr. Stephen Plymate, University of Washington School of Medicine, Seattle donated AR^v567es^ expression plasmid. Mdm2 Monoclonal antibody and polyclonal antibodies against AR N20, ERβ, PR, RARα, RARβ, AR (mouse), calpeptin and ZVAD-fmk were purchased from Santa Cruz. Hsp90, phosphoAR and Hsp70 antibodies were purchased from BD Pharmingen. CHIP monoclonal antibody and MG132 were from Sigma Aldrich, USA. ECL detecting kit was from Thermo Scientific.

### Cell culture and Western blot analysis

Prostate cancer cells were maintained in RPMI supplemented with 10% FBS and 1% penicillin-streptomycin antibiotics (Invitrogen). For experiments using anti-androgens, cells were cultured in phenol red-free RPMI 1640 supplemented with 5% charcoal dextran FBS and 1% penicillin-streptomycin. PWR-1E cells were maintained in Keratinocyte-SFM (1X) with 1 × 2.5 μg EGF human recombinant and 1 × 25 mg bovine pituitary extract (Invitrogen). Cells were lysed with RIPA buffer (Sigma), supplemented with protease inhibitors (Roche), phosphatase inhibitors (Thermo Scientific), 1 mmol/L EDTA and 1 mmol/L PMSF (Sigma). Lysed cells were clarified by pelleting in a table top micro centrifuge at 13300 rpm for 15 min at 4°C. 50–100 μg total cell lysates were denatured in 5X sample buffers and boiled at 99°C for 5 min. Western blotting was performed as previously described [21, 25].

### Immunoprecipitation

LNCaP and CWR22Rv1 were treated with gal (10 μM) for 11 and 14 h to elucidate fAR-mdm2/Hsp90 and AR-V7-CHIP/Hsp70 interaction. In ubiquitination assays, cells were treated with 20 μM gal for 24 h with or without MG132 (5 μM) and lysed with RIPA buffer. 1 mg of total cell lysates were pre-cleared with 30 μl of protein A/G sepharose beads (Santa Cruz), for 45 minutes and pelleted for 1minute at 13300 rpm. Supernatant were collected and incubated with 1 μg of polyclonal antibody per 500 μg of total protein and rotated at 4°C for 12 h. Complexes were washed 3X with lysis buffer and 2X SDS loading dye added to elute proteins prior to separation on a 10% tris/glycine gel.

### Plasmid and siRNA transfections

DU145 cells were transfected with 0.5 μg of AR-V7 or AR^v567es^ expression plasmid, using the Qiagen Effectene transfection reagent following manufacturers’ protocol. Transfection reagents and complexes were washed off 16 h after transfection and replaced with phenol red free media supplemented with 5% charcoal dextran FBS for 20 h. Transfected cells were treated with agents for 24 h and lysed with RIPA buffer. LNCaP and CWR22Rv1 cells were reverse transfected with 100 nM of Mdm2 or CHIP/STUB1 siRNA using lipofectamine RNAiMAX (Invitrogen) for 12 h adhering to manufacturer’s protocol. Scrambled siRNA were transfected as controls. siRNA complexes were replaced with phenol red-free media and treated with agents for 24 h.

### Cell viability (MTT assay) and apoptosis

MTT assays were performed as described in our previous publications [21, 25, 30]. The Acridine orange (AO) and ethidium bromide (EB) (Sigma Aldrich) apoptotic detection assay was used to determine apoptotic cells. Briefly, cells were treated in 6 well plates at 2.5 μM for 72 h. Cells were washed 1X with warm PBS and incubated in 400 μl of 0.1% EB and 0.2% AO in PBS at 37°C for 30 minutes. Cells were again washed once with warm PBS and images taken using fluorescence microscope Nikon TE2000 microscope Flow cytometry analysis was used to detect cell death in CWR22Rv1 cells, using the Moxiflow equipment. Cells were exposed to different concentrations of gal and VNPT55 for 24 h. The Annexin-V-fitc apoptosis detection kit (BD Biosciences) was used following manufacturers protocol.

### RNA isolation and real-time polymerase chain reaction analysis

Cells were seeded in 6-well plates at 0.3 × 10^6^ cells per well and treated with indicated concentrations of compounds for 24 h. RNA was isolated with the Qiagen RNeasy reagents following manufacturer’s protocol. 1800 ng of RNA were reverse transcribed into cDNA using high capacity cDNA reverse conversion kit (life technologies). Full length AR primers, AR-V7 and internal control 18S primer sequences used were as reported in Guo *et al* [16]. Relative expression levels of fAR and AR-V7 were quantified with the comparative ΔΔC_t_ using 18S as internal control.

### Immunocytochemical analysis

CWR22Rv1 cells were seeded and grown to 70% confluence in an 8-chamber slide. Cells were then treated with galeterone and VNPT55 at 5 μM and 10 μM in regular media for 24 h. Treated cells were incubated with 125 nM mitotracker red CMXRos (cell signaling) for 30 minutes. Immunostaining was carried out as previously reported [21]. Alexa fluor 488 conjugated cytochrome c (BD Pharmingen) and Bax antibodies were diluted at 1:500. Images were acquired with the Nikon TE2000 microscope.

### Immunohistochemical analysis

All specimens were kept in 10% buffered formalin for 24 h after which they were embedded in paraffin and 4 μm thick slides prepared and used for immunostaining and hematoxylin-eosin staining. Immunostaining sections were de-parafinised, soaked in alcohol, and antigen retrieval was performed for formalin fixed tissues by heating in citrate buffer (pH = 6). Sections were blocked in 3% hydrogen peroxide for 5 minutes, followed by incubation with the appropriate antibody and epitopes detected using the Ultra-sensitive ABC staining kit (Thermo fisher scientific, USA). The images were captured with an EVOS^®^ FL Auto Imaging System (Life Technologies).

### *In vivo* xenograft tumor growth

All animal studies were performed according to the guidelines approved by the Animal Care Committee of the University Of Maryland, School of Medicine, Baltimore. Male SCID mice 5–6 weeks of age were obtained from the National Cancer Institute (Fredrick, MD). Surgically castrated mice were housed under complete aseptic conditions, fed autoclaved pellets and sterile water ad libitum. Following a week of acclimatization, approximately 5 × 10^6^ CWR22Rv1 cells were inoculated into both flanks. Tumor-bearing mice (tumor volume around 50–70 mm^3^) were randomized into 3 groups (6 mice in each group; compounds formulated in vehicle) and treated as follows: (i) vehicle control (40% β-cyclodextin in ddH_2_O, i.p.), (ii) galeterone (0.15 mmol/kg, i.p., twice daily, 5 days/week) and (iii) VNPT55 (0.15 mmol/kg, i.p., twice daily, 5 days/week). Tumors were measured twice weekly with calipers and tumor volume was calculated by the formula: length × width^2^ × 0.5 (mm^3^). Animals were also weighed weekly and monitored for general health status and signs of possible toxicity due to treatments. Mice were sacrificed after the indicated periods of treatment and tumors and organs excised. Tumors were divided and either flash frozen in liquid nitrogen or placed in 10% buffered formalin for western blot analysis, immunocytochemistry (IHC) and hematoxylin and eosin (H & E) staining.

### Statistical analysis

All *in vitro* experiments were repeated at least three times and reported as means with standard error where applicable. Western blot on *in vivo* samples were repeated in at least 2 different tumor sections from different animals. Student *T*-test and Analysis of variance (ANOVA) were used to determine the significance of deviations or lack thereof.

## SUPPLEMENTARY FIGURES


