# Supplementary material for: Galeterone and VNPT55 induce proteasomal degradation of AR/AR-V7, induce significant apoptosis via cytochrome c release and suppress growth of castration resistant prostate cancer xenografts in vivo
Source: Oncotarget. 2015 Jul 14;6(29):27440–60. doi: 10.18632/oncotarget.4578 (PMC4695001; doi:10.18632/oncotarget.4578)
Supplement: Supplementary file 1 [file oncotarget-06-27440-s001.pdf]

SUPPLEMENTARY FIGURES

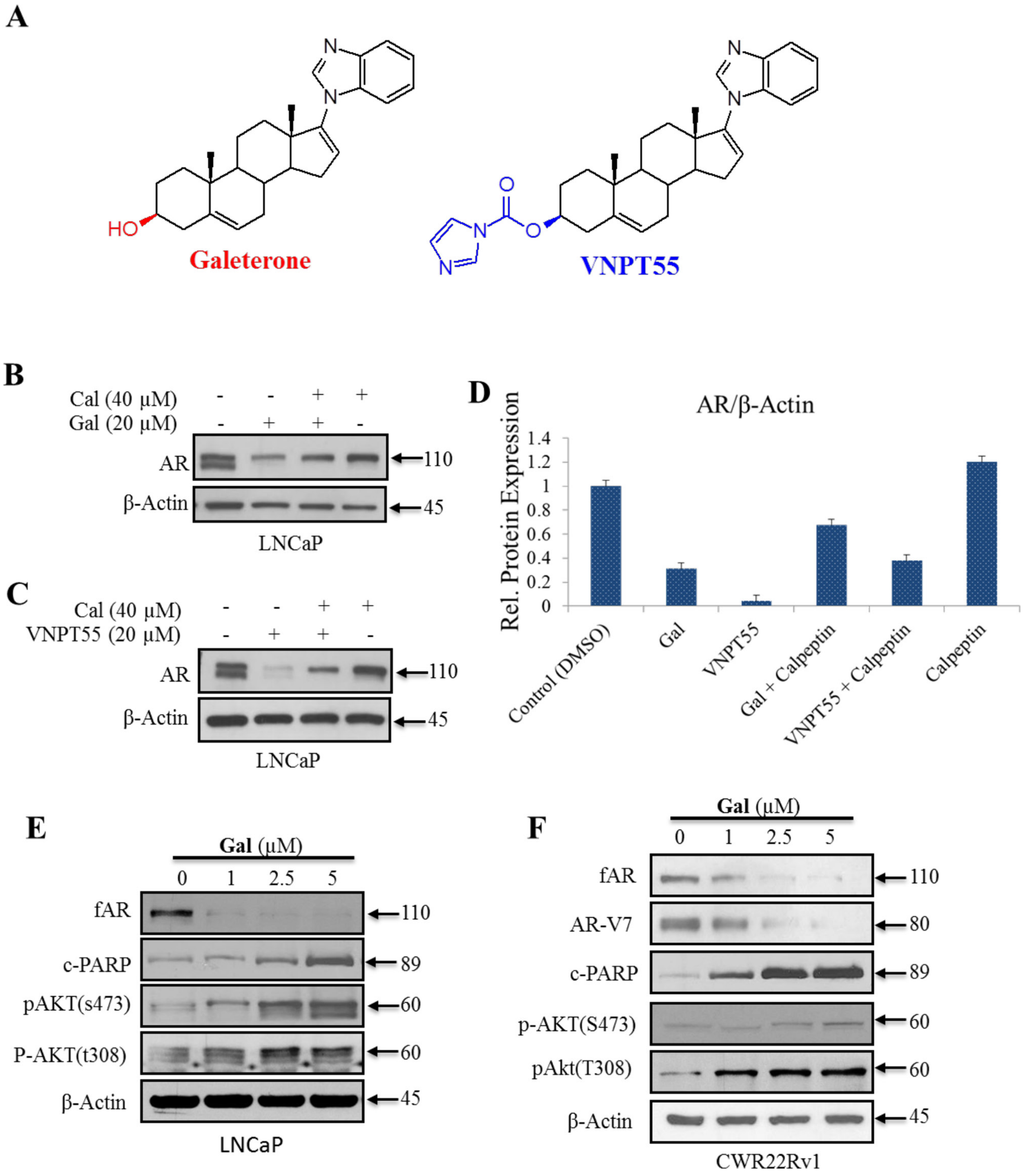

Supplementary Figure S1: Gal/VNPT55 and its effects on AR/AR-V7 protein expression, PARP cleavage and Akt phosphorylation. A. Structures for compounds synthesized in our lab and used in this study (Galeterone and VNPT55). **B.** and **C.** LNCaP cells treated with galeterone and VNPT55 without calpeptin (40 µM) for 24 h and western blot analysis carried out on AR protein expression. Calpain induction serves as a minor pathway for gal and VNPT55 induced fAR degradation. **D.** Densitometry analysis of protein expression in B and C. LNCaP **E.** and CWR22Rv1 **F.** were treated with gal at increasing concentration (1, 2.5 and 5 µM) for 72 h and protein expression of AR, cleaved PARP and phosphorylated forms of Akt analyzed.

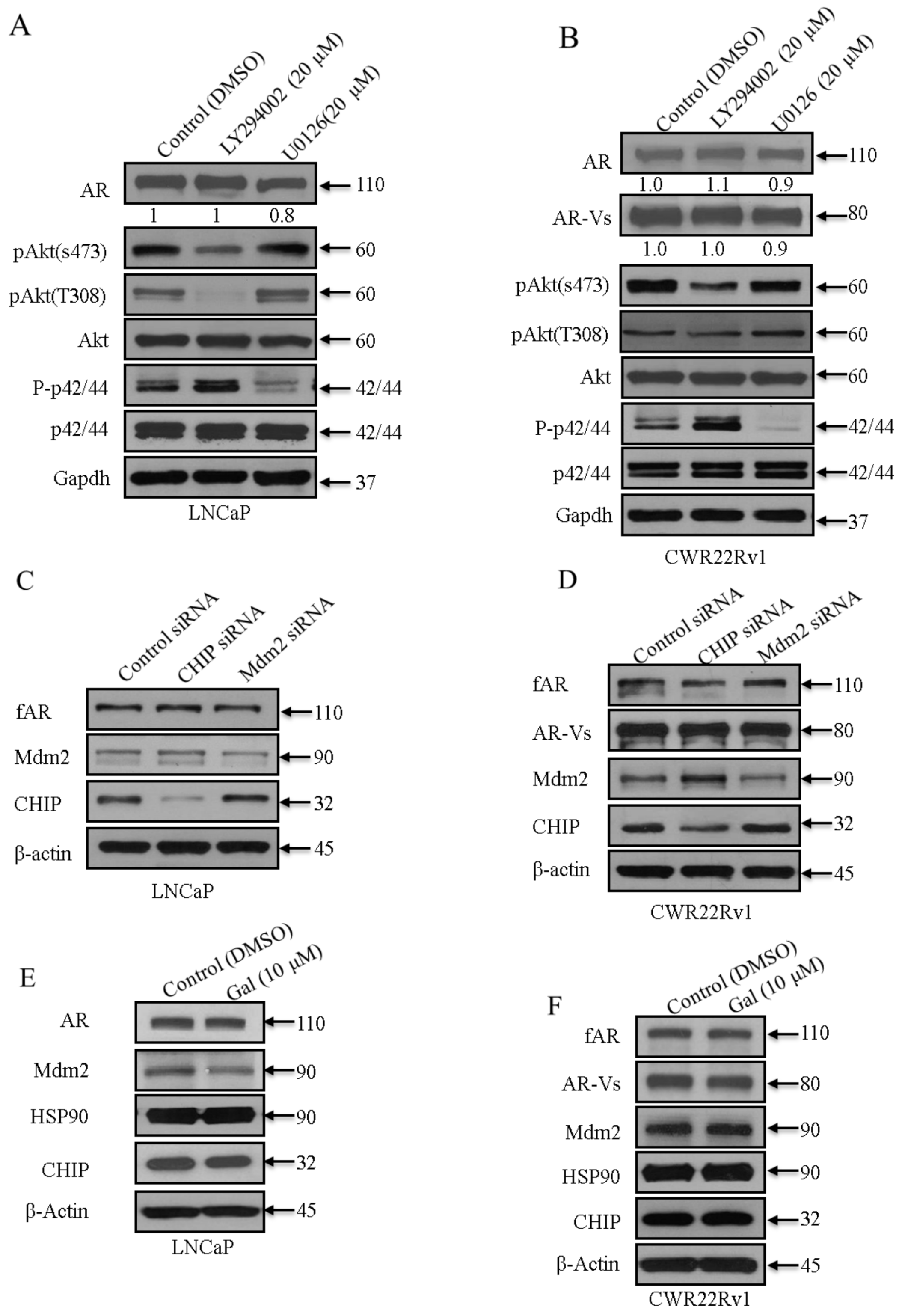

Supplementary Figure S2: Effects on AR/AR-V7 after Mdm2/CHIP knockdown, LY294002, U0126 or gal treatments. **A.** and **B.** LNCaP and CWR22Rv1 cells were treated with PI3k inhibitor (20 μM) and MAPK inhibitor (20 μM) for 24 h and their single effects on AR and AR-V7 analyzed. **C.** and **D.** LNCaP and CWR22Rv1 cells were transfected with Mdm2 and CHIP siRNA for 72 h and effects on fAR/AR-V7 expression levels determined by western blot. **E.** and **F.** Total cell lysates from treated and untreated cells (LNCaP and CWR22Rv1) were analyzed to determine the effects of gal before immunoprecipitation assays were conducted. Protein expression was not significantly altered after gal treatment at the indicated (11 and 14 h) time points to enable equal amounts of immunoprecipitated fAR/AR-V7.
